# Influence of Quinine on the Spleen in Ague

**Published:** 1849-01

**Authors:** 


### ARTICLE III.

### *Influence of Qninine on the Volume of the Spleen in Ague.* (Lancet.)

M. Valleix, physician of the Hôtel Dieu, has directed his attention to the action of the sulphate of quinine on the volume of the spleen in intermittent fever. He has done so to test the accuracy of a statement made by M. Piorry, that the disappearance of the paroxysm coincides with the diminution of the volume of the spleen; that this organ sensibly diminishes in thirty or forty seconds after the administration of a full dose of quinine, in solution, and acidulated; that the diminution goes on very rapidly if the quinine be continued in a sufficiently large dose. M. Gouraud having examined into this matter, however, states that he has not found the spleen thus diminished, but that, in consequence of an accumulation of gas in the stomach, from the injestion of the quinine, the left hypochondrium is rendered sonorous, and the dulness over the spleen becomes masked. These opposite statements M. Valiiex has kept in view in making some fresh observations. He narrates a case, and its course; quite a simple case of ague, occurring in a young and robust man, who had never suffered before. It was a recent case, and there were no evidences of organic diseases in any organ; the spleen had undergone very considerable enlargement, being readily perceived through the abdominal wall, and therefore its size could be estimated with the greatest precision. The sulphate of quinine, although given in a very strong dose of thirty grains, and acidulated, so as to render the salt a bisulphate, did not act, as represented by M. Piorry, on the volume of the spleen, neither at the end of forty seconds, nor of twenty minutes, nor even of twenty-four hours. The medicine also had no such power when given in still greater quantity, but divided, during the day, into several doses, and continued on succeeding days. But after the application of cupping-glasses and leeches over the splenic region, the volume of the spleen, on the contrary, diminished rapidly, although the dose of quinine was abated. Lastly, notwithstanding the persistence of the splenic engorgement, the fever was cut short, and there was no trace of a recurrent paroxysm.

Another equally uncomplicated case occurred to M. Valliex, and the same method being tried, was attended by the same results. It must, however, be mentioned, that three days after the first dose of quinine, a slight diminution of the spleen was noticeable; but this little decrease, which, perhaps, too, was partly owing to a bottle of eau de Vichy, which the patient took, was lost sight of when compared with the rapid diminution which followed two days afterwards, when cupping-glasses were applied over the spleen, and which continued to go on. In this case, also, as in the preceding, although the enlarged spleen remained, the fever was removed.

The third case differed from the two preceding, in that it was of older date; but there was no essential difference in the effects of the treatment. The spleen remained unaffected in size during the first day, when quinine alone was given; but quickly decreased after local bleeding, although the dose of quinine was lessened. The fever was removed before the engorgement of the spleen had subsided.

Thus these observations contradict the assertions of M. Piorry, both as to the coincidence of the disappearance of the fever, and the decrease of the spleen, and as to the immediate and prolonged influence of quinine in diminishing the splenic congestion. M. Valleix also confirms the observation of M. Gouraud as to the formation of gas in the stomach upon the quinine being swallowed, augmenting the resonance over the left hypochondrium, and so hiding the dulness over the solid spleen beneath to a slight extent; not so much so, however, but that palpation and percussion will readily detect the engorged organ.—*Ibid.*

